# Determinants of Early-Stage Breast Cancer Presentation in Western Kazakhstan: A Population-Based Analysis of Urban–Rural Disparities and Diagnostic Pathways (2015–2025)

**DOI:** 10.3390/medicina62081431

**Published:** 2026-07-23

**Authors:** Dinara Zholmukhamedova, Maiya Taushanova, Dariusz Walkowiak, Lyudmila Yermukhanova, Laura Danyarova, Indira Karibayeva, Aizat Aimakhanova, Aizat Seidakhmetova, Anara Tulyayeva

**Affiliations:** 1 Department of Oncology, West Kazakhstan Marat Ospanov Medical University, Aktobe 030019, Kazakhstan; 2 Department of Public Health and Healthcare with a Nursing Course, West Kazakhstan Marat Ospanov Medical University, Aktobe 030019, Kazakhstan; yermukhanova@zkmu.kz; 3 Department of Organization and Management in Health Care, Poznan University of Medical Sciences, 61-701 Poznan, Poland; 4 Republic Center of Endocrinology and Diabetes, Joint Stock Company “Research Institute of Cardiology and Internal Diseases”, Almaty 050000, Kazakhstan; 5 Jiann-Ping Hsu College of Public Health, Georgia Southern University, Statesboro, GA 30458, USA; ik01379@georgiasouthern.edu; 6 Department of Biostatistics and Fundamentals of Scientific Research, Asfendiyarov Kazakh National Medical University, Almaty 050000, Kazakhstan; aimahanova.a@kaznmu.kz; 7 Department of Emergency Medicine and Nursing, Joint Stock Company “South Kazakhstan Medical Academy”, Shymkent 160019, Kazakhstan

**Keywords:** breast cancer, early-stage diagnosis, urban–rural disparity, Kazakhstan, mammography screening, socioeconomic determinants

## Abstract

*Background and Objectives:* Breast cancer is the leading oncological diagnosis among women in Kazakhstan, yet a substantial proportion of cases are still detected beyond the earliest stages, particularly in peripheral regions such as Aktobe. Despite a national mammography screening programme covering women aged 40–70 years since 2018, structural differences in access to early diagnosis—related to geography, socioeconomic circumstances, and the diagnostic pathway—may compromise outcomes. We aimed to identify factors independently associated with early-stage presentation and to characterise the temporal pattern of early-stage presentation without assuming a monotonic trend. *Methods and Materials:* We conducted a retrospective, population-based analytical study of all confirmed breast cancer cases (ICD-10 C50) registered in the Aktobe regional cancer registry and diagnosed between 1 January 2015 and 31 December 2025 (*n* = 2232). The outcome was early-stage presentation, defined literally as Stages I–IIa at diagnosis, with Stages IIb–IV as the comparator; this is a stage-at-presentation classification and is not intended to indicate surgical operability or treatment sequence. Multivariable logistic regression estimated adjusted odds ratios (aORs). Two models were used: Model A included age, sex, residence, administrative nationality, employment/social status, and calendar year; Model B additionally included the diagnostic pathway, which may lie on the causal pathway between structural determinants and stage. Calendar year was modelled as a categorical variable, and a complementary phase-based model (2015–2017, 2018–2019, 2020–2022, 2023–2025) was fitted. A residence-by-year interaction; a multinomial sensitivity analysis separating Stages IIb, III, and IV; discrimination (AUC, Brier score); and calibration (Hosmer–Lemeshow test, calibration plot) were also assessed. *Results:* Of 2232 patients (99.1% female; mean age 57.0 ± 12.5 years), 1323 (59.3%) presented at Stages I–IIa. In Model A, rural residence (aOR 0.77, 95% CI 0.64–0.93; *p* = 0.006), unemployment relative to employment (aOR 0.69, 95% CI 0.52–0.90; *p* = 0.007), Russian administrative nationality (aOR 0.64, 95% CI 0.50–0.82; *p* < 0.001), and other non-Kazakh nationalities (aOR 0.73, 95% CI 0.58–0.94; *p* = 0.012) were independently associated with lower odds of Stage I–IIa presentation. In Model B, patient-initiated (self-referral) presentation was associated with lower odds relative to clinical examination room detection (aOR 0.46, 95% CI 0.30–0.72; *p* < 0.001), whereas organised screening was not significantly associated (aOR 1.52, 95% CI 0.95–2.43; *p* = 0.082). The calendar-year pattern was clearly non-linear (categorical vs. linear year: likelihood-ratio χ^2^ = 76.1, df = 9, *p* < 0.001): odds of Stage I–IIa presentation peaked in 2018 (aOR 2.49, 95% CI 1.57–3.93 vs. 2015), were lowest in 2022 (aOR 0.64, 95% CI 0.42–0.97), and partially recovered thereafter. In the phase-based model (reference 2015–2017), the aORs were 1.62 (95% CI 1.22–2.15) for 2018–2019, 0.54 (95% CI 0.41–0.69) for 2020–2022, and 0.62 (95% CI 0.46–0.84) for 2023–2025. Model discrimination was limited (AUC 0.648, 95% CI 0.625–0.671; Brier score 0.226) with acceptable calibration (Hosmer–Lemeshow χ^2^ = 8.38, df = 8, *p* = 0.40). *Conclusions:* In this registry-based cohort, rural residence, unemployment, non-Kazakh administrative nationality, and patient-initiated presentation were independently associated with lower odds of early-stage breast cancer presentation. The temporal pattern was non-monotonic, with the highest odds around the 2018 screening expansion, a marked reduction during 2020–2022, and only partial recovery thereafter. These are observational associations rather than causal or programme-evaluation findings; they should be interpreted as hypothesis-generating and require confirmation with screening-process, service-capacity, and patient-level access data.

## 1. Introduction

Breast cancer is the most frequently diagnosed malignancy among women worldwide, accounting for approximately 2.3 million new cases and 670,000 deaths in 2022 (GLOBOCAN 2022), with Eastern Asia contributing the largest absolute burden [1,2,3]. In Kazakhstan, breast cancer has been the leading female cancer since 2011; in 2023, approximately 5000 new cases were registered annually, with around 1200 deaths. The age-standardised incidence rate (World Standard) increased from 39.3 per 100,000 women in 2004 to 54.4 in 2023, reflecting a combination of underlying epidemiological changes and increased detection through the national screening programme scale-up [4].

The prognosis of breast cancer is critically dependent on the stage at diagnosis. Five-year survival rates for stage I disease exceed 95% in high-resource settings, declining to below 30% for Stage IV [5,6,7,8]. In this context, the proportion of women diagnosed at early stages (Stages I–IIa) is a widely used indicator of health system performance in oncology. Kazakhstan has addressed this challenge through an organised population-based mammography screening programme: initiated in 2008 for women aged 50–60 years, the programme was substantially expanded in 2018 to cover all women aged 40–70 years, with biennial mammography and BI-RADS classification implemented [4,9,10]. By 2024, reported screening coverage among the target population reached approximately 70% [11].

Yet, national averages mask subnational heterogeneity. Studies from Kazakhstan and other former Soviet Union countries consistently document that rural residents, socioeconomically disadvantaged women, and certain minority groups face multiple intersecting structural barriers to timely cancer detection [11]. These include limited availability of mammography equipment in rural districts, scarce specialist radiologists, transportation challenges in sparsely populated steppe territories, low health literacy, and cultural barriers to screening uptake [12,13,14]. Analysis of national registry data covering 2014–2019 found that urban residents accounted for a disproportionately large share of diagnosed breast cancer cases relative to the national urban population share, a pattern suggestive of differential access to and uptake of diagnostic services [4,15].

The Aktobe region in Western Kazakhstan represents a particularly instructive setting for studying these differences. Covering 299,000 km^2^ with a population of approximately 900,000, it combines a substantial urban centre (Aktobe city) with vast rural and semi-rural areas characterised by oil and gas industry employment alongside traditional pastoralism. The region’s multiethnic composition—predominantly Kazakh, with significant Russian and other minority populations—offers an opportunity to examine both geographic and sociodemographic dimensions of stage at presentation.

Regional analyses of breast cancer in Kazakhstan have shown that stage-specific incidence varies substantially across oblasts, with regions possessing less developed healthcare infrastructure showing higher proportions of advanced-stage disease [4,15]. The Aktobe region has not been the subject of a dedicated population-based study examining the multifactorial determinants of early-stage versus advanced-stage presentation. Understanding which individual and health system factors are associated with early-stage presentation is a necessary prerequisite for designing equity-oriented public health interventions.

The present study addresses this gap by examining all confirmed breast cancer cases diagnosed in the Aktobe region across eleven consecutive calendar years (2015–2025), with the aim of identifying sociodemographic and diagnostic-pathway factors independently associated with Stage I–IIa presentation. We additionally characterise the temporal pattern of stage at presentation without assuming a linear trend and evaluate whether that pattern differs between urban and rural residents. Our findings are intended to inform regional oncology policy, primary care resource allocation, and the design of national screening programmes in Kazakhstan and comparable middle-income settings across Central Asia [16].

## 2. Materials and Methods

### 2.1. Study Design and Setting

This was a retrospective, population-based observational study conducted in the Aktobe region of Western Kazakhstan. The prespecified study period extended from 1 January 2015 to 31 December 2025 and comprised 11 complete calendar years. The Aktobe regional oncology service is the principal publicly funded referral centre for cancer diagnosis and treatment in the region and operates on the clinical base of West Kazakhstan Marat Ospanov Medical University under the Ministry of Healthcare of the Republic of Kazakhstan. Reporting of confirmed cancer diagnoses to the regional oncology registry is mandatory. Regional records are standardised, checked for completeness and internal consistency, and subsequently integrated into the national cancer registration system. These procedures support population-based case ascertainment and the completeness of the registry data used in this study. The study is reported in accordance with the Strengthening the Reporting of Observational Studies in Epidemiology (STROBE) statement for observational studies.

### 2.2. Study Population

The study population comprised all individuals with a histologically or cytologically confirmed malignant neoplasm of the breast, defined using International Classification of Diseases, 10th Revision code C50, who were registered in the Aktobe regional oncology database with a recorded diagnosis date between 1 January 2015 and 31 December 2025.

The study period was defined before data extraction, and records were manually extracted from the registry according to the recorded date of diagnosis. A total of 2232 eligible breast cancer records were identified. All 2232 records had a valid diagnosis date within the prespecified study period, a classifiable disease stage, and complete information for every covariate included in the statistical models. Consequently, no records were excluded because of missing diagnosis date, stage, or covariate information, and the full extracted registry population was included in the analyses. No imputation or other missing-data procedure was required.

Because there were no exclusions between record identification and final analysis, a participant flow diagram was not generated.

Breast cancer predominantly affected women in this cohort, who accounted for 99.1% of all registered cases. The primary analyses included all patients, while a sensitivity analysis restricted to female patients was conducted to assess the stability of the effect estimates.

### 2.3. Outcome Variable

The primary outcome was early-stage presentation, operationalised as a binary variable and defined literally by TNM stage at diagnosis: Stage I or Stage IIa (early-stage) versus Stage IIb, Stage III, or Stage IV (the comparator). Staging was based on TNM clinical or pathological staging as recorded in the registry, and this staging-based definition took precedence over any registry-based early/late classification in cases of discrepancy.

This outcome is a descriptive classification of anatomical disease extent at presentation. Receptor and immunophenotype data were not consistently recorded in the regional registry across the study period and could therefore not be incorporated into the outcome definition; this is addressed in the Limitations (Section 4.8). To avoid relying on a single dichotomisation, a multinomial sensitivity analysis modelling Stage IIb, Stage III, and Stage IV separately against Stages I–IIa was used (Section 2.6).

### 2.4. Explanatory Variables

Covariates were selected a priori on the basis of clinical relevance and prior evidence on disparities in cancer detection.

Age at diagnosis was treated as a continuous variable in years (and as a categorical variable in a sensitivity analysis: <40, 40–49, 50–59, 60–69, and ≥70 years). Sex was recorded as female or male. Residence was categorised as urban or rural on the basis of the registered place of residence at diagnosis. Administrative nationality was recorded in the registry as an administrative category and was grouped as Kazakh, Russian, or Other (Uzbek, Ukrainian, and other nationalities, combined because of small cell counts); it is a documentary attribute and is not equivalent to self-identified ethnicity, language of communication, or cultural identity (Section 4.4). Employment/social status was categorised as employed, retired/with a disability, or unemployed. No individual-level educational attainment variable exists in the registry, and education was therefore not modelled.

Calendar year of diagnosis was modelled as a categorical variable (2015 as reference), and, in a complementary model, as a four-level diagnosis period: 2015–2017 (pre-expansion), 2018–2019 (post-expansion, pre-pandemic), 2020–2022 (pandemic period), and 2023–2025 (post-pandemic period).

Diagnostic pathway reflected the circumstance of detection documented in the registry and was standardised into four categories:

1. Clinical examination room detection—cancer detected during a routine preventive clinical breast/gynaecological examination performed at a primary-care examination room (“smotrovoy kabinet”), in the absence of a patient-reported breast complaint (registry label: “female/male exam room”; this label has been replaced throughout the manuscript);
2. Organised screening—cancer detected through the national mammography screening programme;
3. Patient-initiated/self-referral—the patient presented to medical care because of a self-noticed symptom, most commonly a palpable mass;
4. Other/unspecified—cancer detected during evaluation for an unrelated condition, during hospitalisation, or with the circumstance not specified in the registry.

Because these categories are recorded administratively by the treating service rather than verified against a screening-invitation database, some misclassification—particularly between opportunistic clinical detection, organised screening, and “other/unspecified”—is possible; this is discussed in Section 4.5 and Section 4.8.

### 2.5. Statistical Analysis

Descriptive statistics summarised patient characteristics overall and by stage group. Continuous variables were expressed as means and standard deviations (SD); categorical variables as frequencies and percentages. Between-group differences were assessed with Welch’s two-sample *t*-test for continuous variables and Pearson’s chi-squared test for categorical variables.

Associations with Stage I–IIa presentation were examined using binary logistic regression, and results are expressed as adjusted odds ratios (aORs) with 95% confidence intervals (CIs). Because the mode of detection is plausibly an intermediate variable on the causal pathway between structural determinants (residence, employment, and nationality) and stage at presentation, two models are reported side by side:

− Model A (primary, structural model): age, sex, residence, administrative nationality, employment/social status, and calendar year (categorical). Estimates for the structural determinants in this model represent their total association with stage, including any component operating through the diagnostic pathway.
− Model B (conditional model): Model A additionally adjusted for diagnostic pathway. Estimates for structural determinants in this model are conditional on the pathway; the difference between Models A and B is described descriptively and is not interpreted as a formal mediation analysis.

Calendar year was modelled categorically rather than as a single linear term. The adequacy of a linear year term was tested formally with a likelihood-ratio test comparing the categorical-year model against the corresponding linear-year model. Adjusted annual probabilities of Stage I–IIa presentation with 95% CIs were estimated from Model B using proportionally weighted estimated marginal means and are reported alongside the observed annual proportions. The phase-based model described in Section 2.4 was fitted with the same covariate set as Model B.

Effect modification by residence was assessed in two ways: a global likelihood-ratio test of the residence-by-categorical-year interaction and a secondary, compact model with a residence-by-linear-year interaction term, for which the interaction OR (the multiplicative change in the rural-versus-urban OR per calendar year) with 95% CI is reported.

Model discrimination was quantified using the area under the receiver operating characteristic curve (AUC) with a 95% CI and the Brier score. Calibration was assessed with the Hosmer–Lemeshow goodness-of-fit test, reported with its exact chi-square statistic, degrees of freedom, and *p*-value, and with a decile-based calibration plot. Multicollinearity was evaluated using generalised variance inflation factors (GVIFs). All analyses were performed in R version 4.5.1 (RStudio 2025.9.0.387), with a two-sided significance threshold of *p* < 0.05.

### 2.6. Sensitivity Analyses Methods

Five sensitivity analyses were conducted: (1) a multinomial logistic regression with Stages I–IIa as the reference category and Stage IIb, Stage III, and Stage IV as separate outcomes; (2) a phase-based temporal model; (3) restriction of the outcome definition to Stage I only; (4) restriction of the analytical sample to female patients; and (5) replacement of continuous age with categorical age groups.

### 2.7. Ethics

This study was approved by the Local Ethics Committee of Marat Ospanov West Kazakhstan Medical University (protocol №1. 1-2026/004-D, 30 January 2026). As a retrospective analysis of routinely collected registry data, individual informed consent was not required under applicable national regulatory provisions.

## 3. Results

### 3.1. Sample Characteristics

The cohort was almost exclusively female (*n* = 2213, 99.1%), with 19 male cases (0.9%). Mean age at diagnosis was 57.0 years (SD 12.5). Most patients were urban residents (*n* = 1481, 66.4%) and of Kazakh administrative nationality (*n* = 1504, 67.4%); 368 (16.5%) were Russian and 360 (16.1%) of other nationalities. Regarding employment/social status, 1005 (45.0%) were employed, 933 (41.8%) retired or with a disability, and 294 (13.2%) unemployed. Patient-initiated/self-referral was the most common diagnostic pathway (*n* = 1034, 46.3%), followed by other/unspecified circumstances (*n* = 711, 31.9%), organised screening (*n* = 363, 16.3%), and clinical examination room detection (*n* = 124, 5.6%).

The stage distribution was Stage I, 504 (22.6%); Stage IIa, 819 (36.7%); Stage IIb, 523 (23.4%); Stage III, 273 (12.2%); and Stage IV, 113 (5.1%). Overall, 1323 patients (59.3%) presented at Stages I–IIa and 909 (40.7%) at Stages IIb–IV. Baseline characteristics stratified by stage group are presented in Table 1.

### 3.2. Multivariable Models

**Figure 1:**
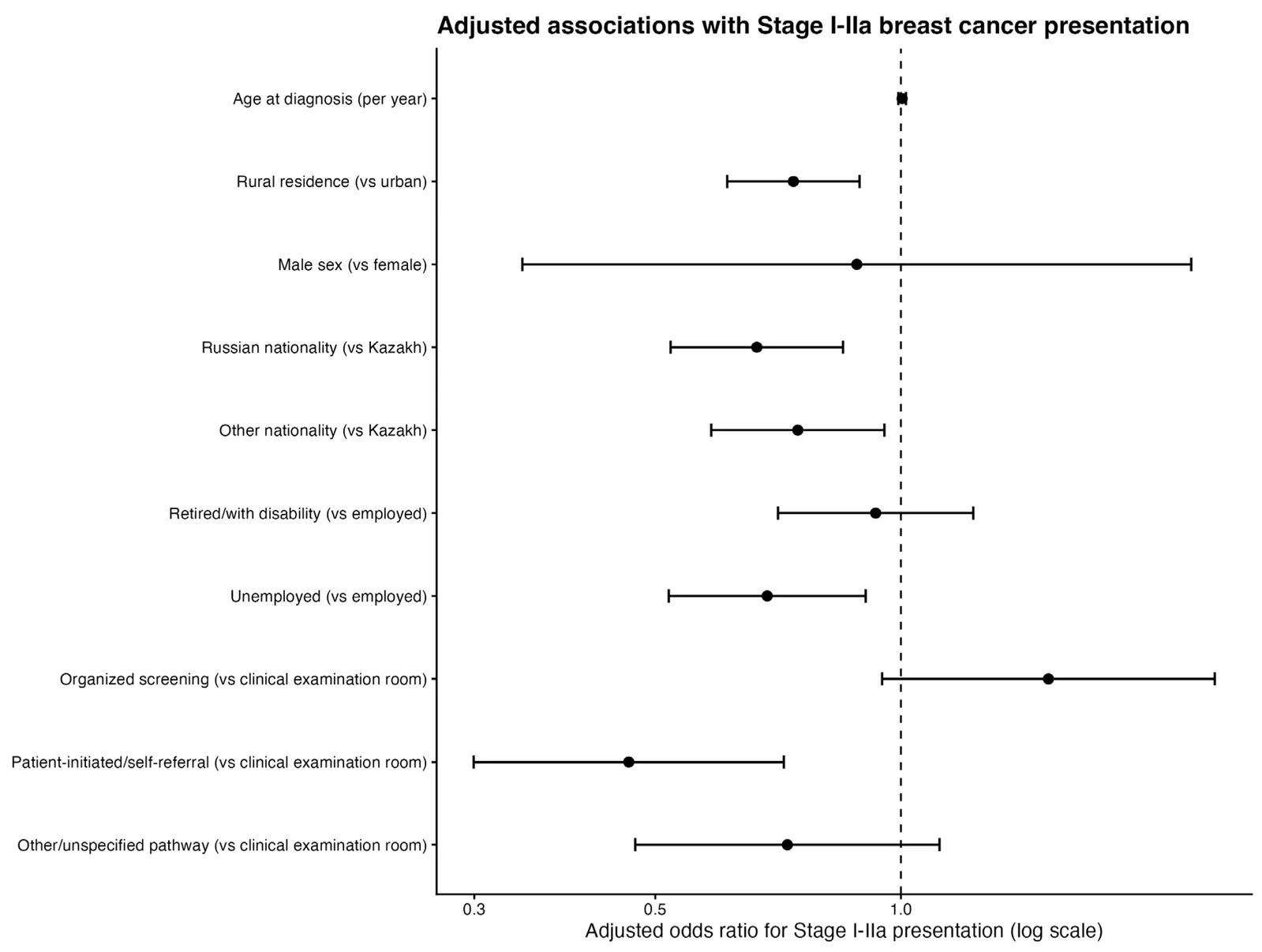
Forest plot of adjusted odds ratios for Stage I–IIa breast cancer presentation (Model B), Aktobe region, 2015–2025. Points represent aORs; horizontal bars represent 95% CIs; the dashed vertical line indicates the null value of 1.0.

Results of the two multivariable models are presented in Table 2 and Figure 1. In Model A, which did not include the diagnostic pathway, rural residence was independently associated with lower odds of Stage I–IIa presentation compared with urban residence (aOR 0.77, 95% CI 0.64–0.93; *p* = 0.006). Compared with Kazakh patients, patients recorded as having Russian administrative nationality (aOR 0.64, 95% CI 0.50–0.82; *p* < 0.001) or another non-Kazakh administrative nationality (aOR 0.73, 95% CI 0.58–0.94; *p* = 0.012) had lower odds of Stage I–IIa presentation. Unemployment was also associated with lower odds relative to employment (aOR 0.69, 95% CI 0.52–0.90; *p* = 0.007), whereas retired patients or those with a disability did not differ significantly from employed patients (aOR 0.92, 95% CI 0.70–1.20; *p* = 0.526). Age (aOR 1.01 per year, 95% CI 0.99–1.02; *p* = 0.350) and male sex (aOR 0.78, 95% CI 0.31–2.00; *p* = 0.612) were not significantly associated with the outcome, although statistical power for the sex comparison was very limited.

**Table 2:** Factors associated with Stage I–IIa breast cancer presentation, Aktobe region, 2015–2025.

Characteristic	Model A: aOR (95% CI)	*p*-Value	Model B: aOR (95% CI)	*p*-Value	GVIF^(1/(2 × df))
Age at diagnosis (per year)	1.01 (0.99, 1.02)	0.350	1.00 (0.99, 1.01)	0.524	1.55
Rural residence (vs. urban)	0.77 (0.64, 0.93)	0.006	0.74 (0.61, 0.89)	0.001	1.02
Male sex (vs. female)	0.78 (0.31, 2.00)	0.612	0.88 (0.34, 2.27)	0.796	1.01
Russian nationality (vs. Kazakh)	0.64 (0.50, 0.82)	<0.001	0.67 (0.52, 0.85)	0.001	1.02
Other nationality (vs. Kazakh)	0.73 (0.58, 0.94)	0.012	0.75 (0.59, 0.95)	0.020	
Retired/with a disability (vs. employed)	0.92 (0.70, 1.20)	0.526	0.93 (0.71, 1.23)	0.612	1.26
Unemployed (vs. employed)	0.69 (0.52, 0.90)	0.007	0.69 (0.52, 0.91)	0.008	
Organised screening (vs. clinical examination room)	—	—	1.52 (0.95, 2.43)	0.082	1.18
Patient-initiated/self-referral (vs. clinical examination room)	—	—	0.46 (0.30, 0.72)	<0.001	
Other/unspecified pathway (vs. clinical examination room)	—	—	0.73 (0.47, 1.12)	0.144	

Model A was adjusted for age, sex, residence, administrative nationality, employment/social status, and calendar year. Model B was additionally adjusted for diagnostic pathway. Calendar year was included as a categorical variable in both models (2015 as reference); year-specific estimates are shown in Table 3 and Supplementary Table S1. Abbreviations: CI = Confidence Interval, OR = Odds Ratio.

**Table 3:** Observed and adjusted annual probability of Stage I–IIa presentation, Aktobe region, 2015–2025.

Year	Total Cases	Stage I–IIa Cases	Observed Proportion (%)	Adjusted Probability, % (95% CI)
2015	159	88	55.3	62.1 (54.2–69.4)
2016	158	94	59.5	66.1 (58.4–73.1)
2017	149	95	63.8	71.7 (63.9–78.4)
2018	194	147	75.8	81.7 (75.8–86.4)
2019	252	172	68.3	72.9 (66.8–78.3)
2020	157	84	53.5	61.9 (53.5–69.7)
2021	220	112	50.9	52.6 (45.7–59.4)
2022	213	98	46.0	40.1 (33.4–47.1)
2023	265	146	55.1	45.1 (38.3–52.1)
2024	230	132	57.4	51.9 (44.6–59.2)
2025	235	155	65.9	56.4 (48.9–63.6)

Adjusted probabilities were derived from Model B using proportionally weighted estimated marginal means. Abbreviation: CI = confidence interval.

In Model B (additionally adjusted for the diagnostic pathway), the structural associations remained broadly similar: rural residence (aOR 0.74, 95% CI 0.61–0.89; *p* = 0.001), Russian administrative nationality (aOR 0.67, 95% CI 0.52–0.85; *p* = 0.001), other non-Kazakh administrative nationalities (aOR 0.75, 95% CI 0.59–0.95; *p* = 0.020), and unemployment (aOR 0.69, 95% CI 0.52–0.91; *p* = 0.008) remained associated with lower odds of Stage I–IIa presentation. Relative to clinical examination room detection, patient-initiated/self-referral presentation was associated with substantially lower odds of Stage I–IIa presentation (aOR 0.46, 95% CI 0.30–0.72; *p* < 0.001), while organised screening (aOR 1.52, 95% CI 0.95–2.43; *p* = 0.082) and other/unspecified circumstances (aOR 0.73, 95% CI 0.47–1.12; *p* = 0.144) did not reach statistical significance.

Generalised variance inflation factors were low for all predictors (GVIF^1⁄(2·df)^ ≤ 1.55), indicating no evidence of problematic multicollinearity.

### 3.3. Temporal Pattern in Stage I–IIa Presentation

The assumption of a linear calendar-year trend was not supported by the data: a model with categorical year fitted significantly better than a model with a single linear year term (likelihood-ratio χ^2^ = 76.09, df = 9, *p* < 0.001). All temporal results are therefore reported using categorical year, and the previously reported single per-year odds ratio is not presented, as it would misrepresent a clearly non-monotonic pattern.

**Figure 2:**
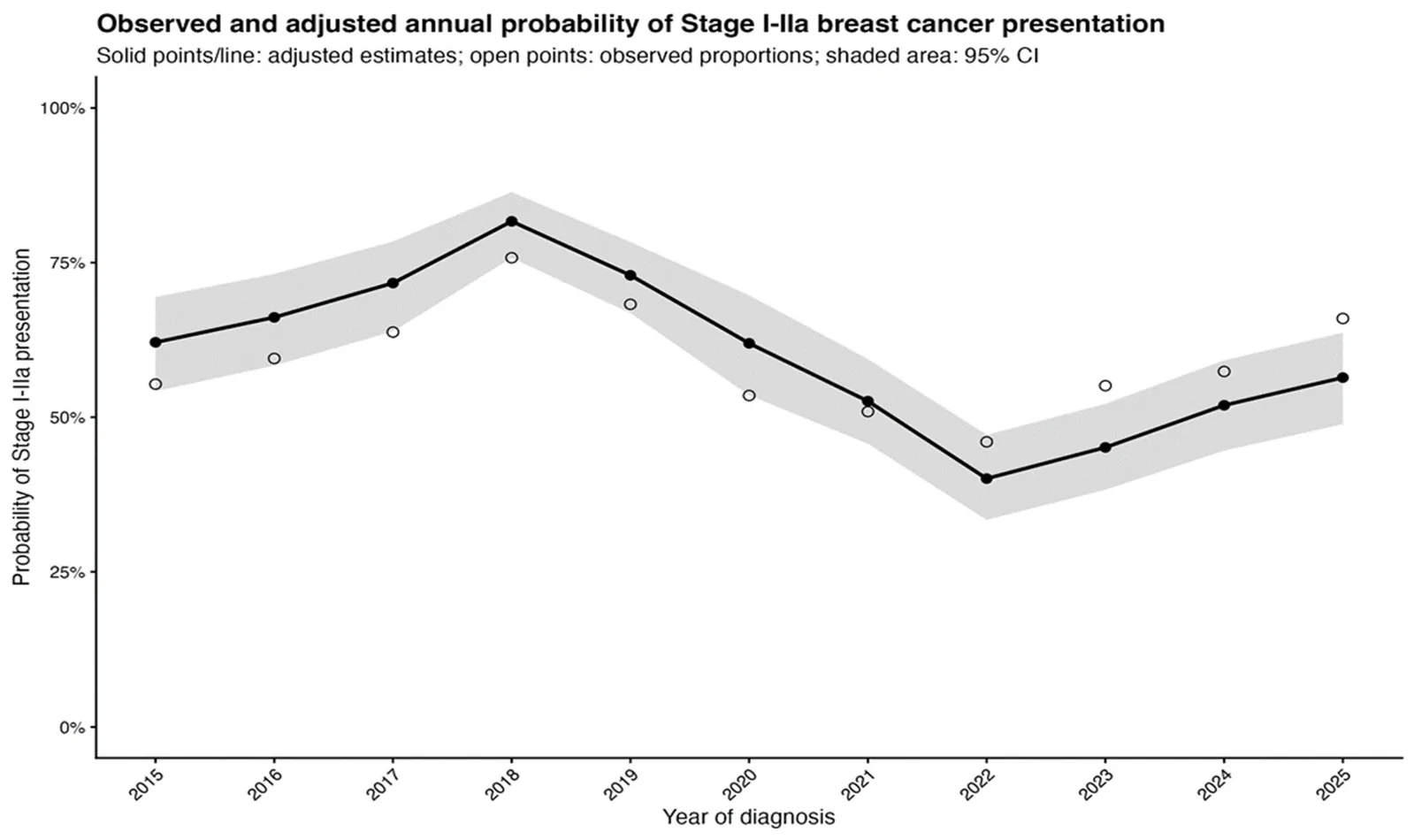
Observed and adjusted annual probability of Stage I–IIa breast cancer presentation, Aktobe region, 2015–2025. Calendar year was modelled categorically; error bars represent 95% CIs for the adjusted probabilities. The figure is descriptive and does not attribute the observed pattern to any single cause.

Observed and adjusted annual probabilities of Stage I–IIa presentation are shown in Table 3 and Figure 2. The observed proportion rose from 55.3% in 2015 to a peak of 75.8% in 2018, fell to a minimum of 46.0% in 2022, and partially recovered to 65.9% in 2025. Relative to 2015, the adjusted odds of Stage I–IIa presentation were highest in 2018 (aOR 2.49, 95% CI 1.57–3.93; *p* < 0.001) and 2019 (aOR 1.71, 95% CI 1.13–2.58; *p* = 0.011), and lowest in 2022 (aOR 0.64, 95% CI 0.42–0.97; *p* = 0.036); the estimates for 2023–2025 did not differ significantly from 2015 in Model A (2025: aOR 1.44, 95% CI 0.95–2.19; *p* = 0.088).

The phase-based model was consistent with this pattern (Supplementary Table S1). Relative to 2015–2017, the adjusted odds of Stage I–IIa presentation were higher in 2018–2019 (aOR 1.62, 95% CI 1.22–2.15; *p* < 0.001), lower during 2020–2022 (aOR 0.54, 95% CI 0.41–0.69; *p* < 0.001), and remained below the pre-expansion reference in 2023–2025 (aOR 0.62, 95% CI 0.46–0.84; *p* = 0.002). The year-by-year stage distribution is shown in Supplementary Figure S1 and Supplementary Table S2.

### 3.4. Residence and the Temporal Pattern

**Figure 3:**
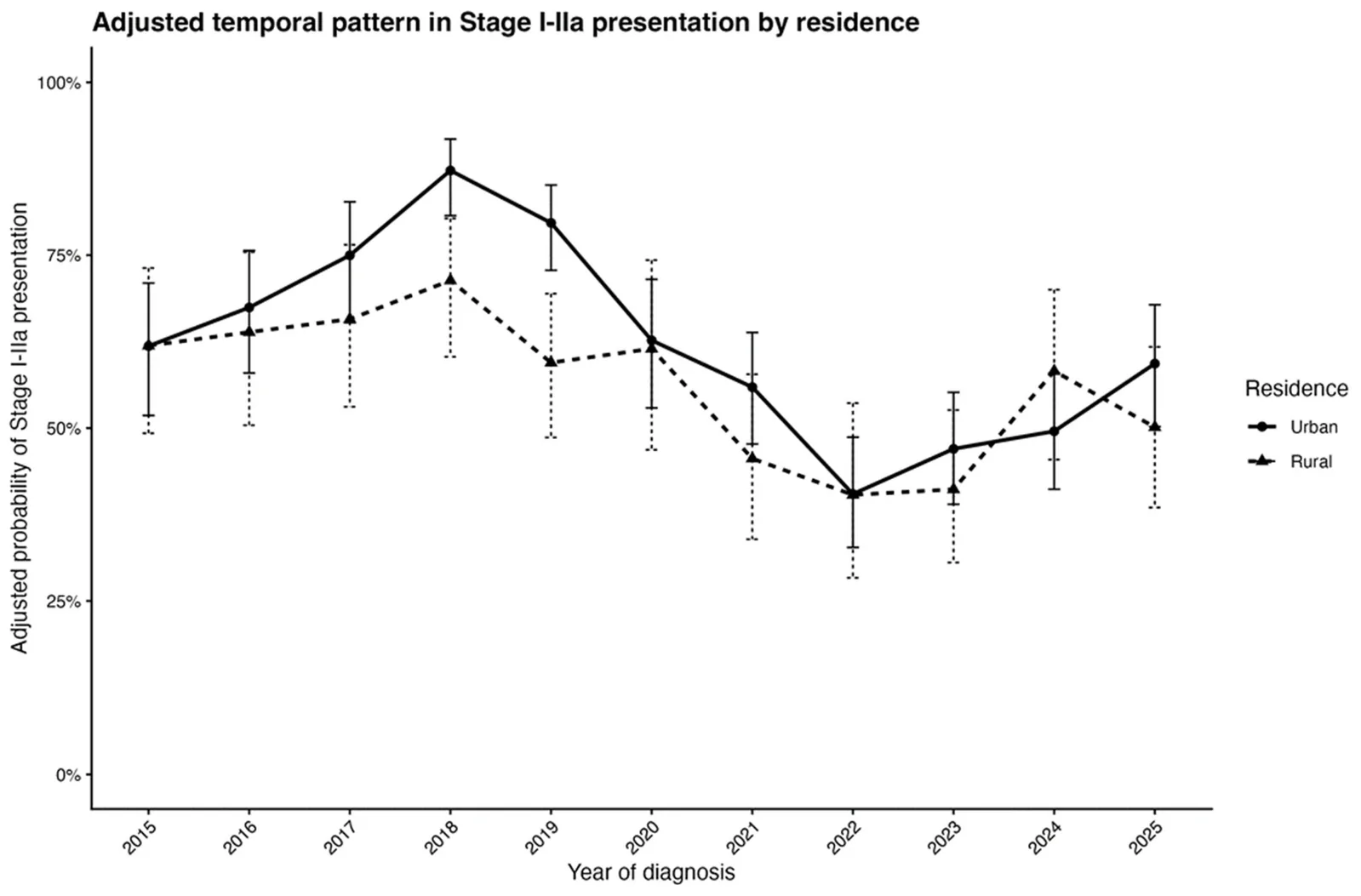
Adjusted annual probability of Stage I–IIa breast cancer presentation by residence, Aktobe region, 2015–2025 (with 95% CIs).

Urban patients had a higher probability of Stage I–IIa presentation than rural patients in most years (Figure 3). However, there was no statistically significant evidence that the temporal pattern differed by residence: the global likelihood-ratio test for the residence-by-categorical-year interaction was not significant (χ^2^ = 17.52, df = 10, *p* = 0.064; Supplementary Table S3), and the secondary compact model estimated the multiplicative change in the rural-versus-urban odds ratio per calendar year as 1.04 (95% CI 0.98–1.10; *p* = 0.206; Supplementary Table S4). The urban–rural difference therefore appears broadly stable across the study period rather than widening or narrowing, although this study is not powered to exclude modest effect modification. Adjusted annual probabilities by residence are shown in Supplementary Table S5.

### 3.5. Model Performance

Model B showed limited discrimination (AUC 0.648, 95% CI 0.625–0.671; Brier score 0.226; Supplementary Table S6 and Supplementary Figure S3). Calibration was acceptable: the Hosmer–Lemeshow test was not significant (χ^2^ = 8.38, df = 8, *p* = 0.397), and the decile-based calibration plot showed close agreement between predicted and observed probabilities across the range of predicted risk (mean predicted 37.8–80.4% vs. observed 39.7–80.3%; Supplementary Table S7 and Supplementary Figure S2). The model is presented as a population-level association model and is not intended, and should not be used, for individual-level prediction.

### 3.6. Sensitivity Analyses Results

Multinomial model (Supplementary Table S8). With Stages I–IIa as the reference category, the associations were not uniform across the advanced-stage categories. Rural residence was associated most strongly with Stage III (adjusted relative risk ratio [RRR] 1.74, 95% CI 1.32–2.30; *p* < 0.001), with weaker and non-significant associations for Stage IIb (RRR 1.23, 95% CI 0.98–1.54) and Stage IV (RRR 1.08, 95% CI 0.70–1.67). Russian nationality was associated with all three categories, most strongly with Stage IV (RRR 2.00, 95% CI 1.21–3.30; *p* = 0.007). Unemployment was associated specifically with Stage IV (RRR 2.22, 95% CI 1.25–3.93; *p* = 0.006). Patient-initiated presentation was strongly associated with Stage III (RRR 4.58, 95% CI 1.90–11.04) and Stage IV (RRR 3.95, 95% CI 1.49–10.47) but not with Stage IIb (RRR 1.38, 95% CI 0.83–2.29), and organised screening was inversely associated with Stage IV (RRR 0.06, 95% CI 0.01–0.30). These results indicate that the binary Stage I–IIa versus Stage IIb–IV contrast conceals clinically meaningful heterogeneity and that the structural determinants relate primarily to locally advanced and metastatic disease rather than to Stage IIb. The Stage IV estimate for male sex was unstable because of complete separation (no male Stage IV cases) and should not be interpreted.

Female-only and categorical-age models (Supplementary Tables S9 and S10). Restriction to female patients produced virtually identical estimates (rural residence aOR 0.74, 95% CI 0.61–0.89; unemployment aOR 0.68, 95% CI 0.52–0.90; patient-initiated presentation aOR 0.44, 95% CI 0.28–0.69). With categorical age, the main associations were also unchanged, and women aged 50–59 (aOR 1.44, 95% CI 1.03–2.02) and 60–69 years (aOR 1.66, 95% CI 1.06–2.61) had higher odds of Stage I–IIa presentation than women aged <40 years, consistent with the higher proportion of advanced-stage disease observed among the youngest patients.

When the outcome was restricted to Stage I versus Stages IIa–IV (Supplementary Table S11), the association with other non-Kazakh nationalities (aOR 0.66, 95% CI 0.48–0.90; *p* = 0.009) and with patient-initiated presentation (aOR 0.55, 95% CI 0.34–0.89; *p* = 0.015) persisted, while the associations with rural residence (aOR 0.98, 95% CI 0.79–1.23; *p* = 0.883), unemployment (aOR 0.79, 95% CI 0.54–1.16; *p* = 0.233), and Russian nationality (aOR 0.74, 95% CI 0.55–1.01; *p* = 0.059) were attenuated and no longer statistically significant. This indicates that the residence and employment associations operate mainly at the Stage IIa/IIb boundary and in more advanced disease rather than at the Stage I threshold, and it is reported here as a limitation of robustness rather than as confirmation of the primary model.

## 4. Discussion

### 4.1. Principal Findings

In this population-based analysis of 2232 breast cancer patients diagnosed in the Aktobe region across eleven calendar years, four findings emerge. First, rural residence was independently associated with approximately 23% lower odds of Stage I–IIa presentation, indicating a persisting geographic gradient in stage at diagnosis [17]. Second, unemployment—but not retirement or disability—was associated with lower odds of early-stage presentation, implicating precarious socioeconomic circumstances rather than age-related disadvantage per se [18]. Third, patients of Russian and other non-Kazakh administrative nationality had lower odds of early-stage presentation than Kazakh patients [18,19]. Fourth, the temporal pattern was non-monotonic: the highest odds of early-stage presentation were observed around 2018–2019, coinciding with the national expansion of mammographic screening, followed by a marked reduction during 2020–2022 and only partial recovery in 2023–2025 [20,21]. We emphasise at the outset that all of these findings are observational associations from registry data. They do not establish causal relationships; do not constitute an evaluation of the national screening programme; and are best regarded as hypothesis-generating signals for targeted, prospectively designed investigation.

### 4.2. The Rural–Urban Disparity

The association between rural residence and later-stage presentation is consistent with findings from elsewhere in Kazakhstan and the wider region. National registry analyses have documented that urban patients disproportionately access both screening and specialist oncology services [4], and regional studies indicate that the availability of mammography equipment, experienced radiologists, and multidisciplinary oncology teams is lower in peripheral districts than in major urban centres [22]. The Aktobe region, despite hosting a major city, encompasses extensive rural territories where physical distance to diagnostic services, limited transport infrastructure, and sparse primary care networks may collectively impair timely detection [17].

These structural factors may be compounded by demand-side factors, including lower health literacy, cultural acceptability concerns around breast examination, and the perception among rural women that screening is less accessible or relevant to them [23,24,25,26]. It is notable that in the multinomial analysis, the rural association was concentrated in Stage III disease rather than distributed evenly across advanced stages, which is compatible with delayed presentation of locally advanced tumours. Because our data contain neither individual screening histories nor distance-to-facility measures, these mechanisms remain hypotheses; testing them requires linkage of registry data to screening-invitation and service-capacity data.

### 4.3. Socioeconomic Determinants and the Role of Employment

The independent association between unemployment and later-stage presentation (aOR 0.69) requires careful interpretation. In Kazakhstan, employed individuals are more likely to attend periodic occupational health examinations and scheduled dispensarisation visits, which may increase opportunities for clinical breast examination and screening referral [27]. Employed women may also have greater social support, higher baseline health literacy, and better logistical capacity to attend appointments [28], whereas unemployed women may face financial and social stressors that compete with preventive care [17].

The absence of a significant difference between retired/disabled and employed patients is consistent with the hypothesis that structured, recurring contact with the health system—rather than economic status alone—is the relevant mechanism, since this group is actively targeted by pension-linked dispensarisation [29]. We note, however, that the association with unemployment was concentrated in Stage IV disease in the multinomial model and was attenuated when the outcome was restricted to Stage I, so it should not be over-interpreted. Whether social insurance and welfare registration status affects detection outcomes for unemployed women is a question that our data cannot answer and that merits dedicated study.

### 4.4. Administrative Nationality

Patients of Russian and other non-Kazakh administrative nationality had lower odds of early-stage presentation than Kazakh patients, and this association persisted across the female-only, categorical-age, and pathway-adjusted models. We consider this a potentially important signal, but we deliberately refrain from causal or cultural interpretation.

The registry variable is administrative nationality—a documentary attribute—and is not equivalent to self-identified ethnicity, first language, cultural identity, religiosity, or trust in the healthcare system, none of which was measured in this study. The observed association may reflect residual confounding by geography (differential settlement patterns and district-level service availability), socioeconomic circumstances not captured by the three-level employment variable, language of healthcare communication, household composition, or differential access to and uptake of screening [30,31,32]. Published survey work from Kazakhstan reporting differences in trust in healthcare professionals between Kazakh-speaking and Russian-speaking respondents [33] is compatible with one such mechanism, but that study did not demonstrate a link to healthcare utilisation, and we cannot test any mediating pathway with the present data.

Accordingly, we frame this finding as hypothesis-generating. It warrants disaggregation of the heterogeneous “Other” category and direct measurement of language, geography, socioeconomic position, and healthcare access in future work, and its inclusion in equity-monitoring frameworks should be understood as a prompt for investigation rather than as evidence of an ethnocultural barrier.

### 4.5. Diagnostic Pathways and Their Interpretation

The inverse association between patient-initiated presentation and early-stage disease (aOR 0.46 relative to clinical examination room detection) is clinically coherent: self-referring patients have typically already noticed a symptom, most often a palpable mass, and symptomatic tumours are larger and more advanced than screen-detected or incidentally detected subclinical lesions [34,35]. The point estimate for organised screening was above unity but did not reach statistical significance (aOR 1.52, 95% CI 0.95–2.43), which is unsurprising given that only 16.3% of cases in this cohort were ascertained through that pathway; in the multinomial analysis, screening was, however, strongly and inversely associated with Stage IV disease.

Two interpretative cautions are essential. First, the mode of detection is plausibly an intermediate variable rather than a simple confounder: residence, employment, and nationality may influence stage at presentation partly through the pathway by which a cancer is found. For this reason, we report both an unconditional structural model (Model A) and a pathway-conditional model (Model B); the near-identical structural estimates across the two models indicate that the recorded pathway does not explain away the structural associations, but this descriptive comparison is not a formal mediation analysis and does not identify a mediated effect. Second, the pathway categories are recorded administratively and were not verified against a screening-invitation database, so misclassification—particularly between opportunistic clinical detection, organised screening, and “other/unspecified”—is possible and would tend to attenuate the contrasts between pathways.

With these caveats, the pattern is consistent with the value of structured clinical breast examination during routine primary care contacts [36,37] and with the need to increase the share of cases identified through organised screening [7,37], but it does not by itself establish that either intervention would change stage distribution at the population level.

### 4.6. The Temporal Pattern

Our reanalysis shows that a single linear calendar-year term is not an adequate description of these data (likelihood-ratio χ^2^ = 76.09, df = 9, *p* < 0.001). Rather than a uniform year-on-year decline, the data show a rise in early-stage presentation to a peak in 2018, a substantial fall during 2020–2022, and partial recovery in 2023–2025, with the post-pandemic level in the pathway-adjusted phase model still below the 2015–2017 reference (aOR 0.62, 95% CI 0.46–0.84).

Several explanations are plausible and not mutually exclusive. The 2018 peak coincides temporally with the expansion of the national mammography programme to women aged 40–70 years, although our design cannot attribute the peak to that expansion. The 2020–2022 reduction coincides with the COVID-19 pandemic, during which cancer screening activity was disrupted in Kazakhstan and internationally, with delayed presentations shifting stage distributions [21,38]. Increasing case volumes from an ageing population may have strained diagnostic capacity [4,39]; compositional change in the case mix could shift the population-level proportion even without changes in system performance [6]; and improving completeness of registry staging over time could contribute a data artefact [7]. Distinguishing between these explanations requires screening-process indicators (invitation, attendance, recall, and interval-cancer data), service-capacity data, and time-to-diagnosis intervals, none of which are available in the present registry extract.

We therefore describe the pattern rather than diagnose its cause. In particular, we no longer characterise the temporal pattern as a “public health failure” or as evidence of a screening-quality deficit: the data are compatible with such an interpretation but do not establish it. What the data do support is that stage at presentation in this region has not recovered to its 2018–2019 level, which is a reasonable trigger for programme-level evaluation and for routine monitoring of stage distribution as a performance indicator [40,41].

### 4.7. Comparison with National and International Data

The overall Stage I–IIa proportion of 59.3% is broadly consistent with previously reported Kazakhstani figures, although national averages mask regional heterogeneity, with a more favourable stage shift documented in major urban centres than in peripheral regions [4,42]. Internationally, early-stage proportions of 70–80% are routinely reported in high-income countries with mature organised screening systems [7], which indicates the size of the gap that remains. The socioeconomic and geographic patterns we observe parallel findings from other post-Soviet health systems and from middle-income countries in Asia [31,43], where the consistent message is that programme infrastructure alone is not sufficient for equitable early diagnosis [43].

The model’s limited discrimination (AUC 0.648, 95% CI 0.625–0.671) is expected for a model built exclusively from administrative and sociodemographic predictors without tumour biological variables (receptor status, grade, tumour size), imaging characteristics, or individual screening history [7,22,34,44]. Calibration was acceptable (Hosmer–Lemeshow *p* = 0.40), meaning that the model reproduces group-level probabilities reasonably well, but discrimination of this magnitude is insufficient for individual-level prediction. The model’s value lies in quantifying independent, population-level associations of potentially actionable structural factors with stage at presentation.

### 4.8. Strengths and Limitations

This study has several important strengths. It includes nearly all registered breast cancer cases in the Aktobe region over eleven consecutive calendar years and contains no missing data for the variables included in the models. The outcome was defined according to stage at diagnosis rather than treatment strategy, and the diagnostic pathway was considered separately because it may represent an intermediate factor between structural characteristics and stage at presentation. Calendar time was analysed without assuming a linear trend, and the robustness of the findings was examined in several sensitivity analyses, including a multinomial model that considered Stage IIb, Stage III, and Stage IV separately.

The findings should nevertheless be interpreted in light of several limitations. The regional registry did not consistently contain information on tumour subtype, grade, hormone receptor and HER2 status, Ki-67, tumour size, individual screening history, family history, body mass index, comorbidities, or distance to diagnostic services. Some of these factors may be related both to stage at presentation and to the likelihood of detection; therefore, residual confounding remains possible.

The binary outcome of Stages I–IIa versus Stages IIb–IV should also be interpreted as a summary measure of disease extent rather than as an indicator of operability or treatment sequence. The multinomial analysis showed that associations were not uniform across Stage IIb, Stage III, and Stage IV, while the Stage-I-only analysis indicated that the associations with residence and employment were less consistent when a more restrictive definition was used.

Several explanatory variables were derived from administrative records and may not fully represent the underlying concepts. Administrative nationality does not capture self-identified ethnicity, language, or cultural identity [32]; registered residence does not directly measure geographic accessibility [6]; and employment status provides only a limited indication of socioeconomic position. Educational attainment was not available. Similarly, the diagnostic pathway was recorded administratively and could not be verified using screening invitation or attendance records, so some misclassification is possible.

Finally, the retrospective observational design does not allow causal conclusions. Survival and cause-specific mortality data were not available, and it was therefore not possible to determine whether the observed differences in stage at presentation were associated with subsequent survival. The temporal findings are also descriptive because the registry did not include detailed information on screening activity, diagnostic capacity, waiting times, or changes in case mix. Consequently, the relative contribution of programme performance, pandemic-related disruption, changes in the patient population, and registry practices could not be determined.

## 5. Conclusions

In this population-based analysis, rural residence, unemployment, non-Kazakh administrative nationality, and patient-initiated presentation were independently associated with lower odds of Stage I–IIa breast cancer presentation in the Aktobe region between 2015 and 2025. The temporal pattern was non-monotonic, with the highest odds of early-stage presentation around the 2018 screening expansion, a marked reduction during the 2020–2022 pandemic period, and incomplete recovery thereafter.

These findings are associations derived from routinely collected registry data and should not be read as causal statements, as an evaluation of the national screening programme, or as evidence of an ethnocultural barrier. They do, however, identify specific groups and time periods in which stage at presentation is less favourable, and they suggest priorities for further investigation and for programme-level evaluation: (1) assessment of screening reach, attendance, and quality in rural districts, including the potential role of mobile mammography and community health worker facilitation; (2) evaluation of engagement strategies for unemployed and socioeconomically vulnerable women; (3) direct measurement of the geographic, linguistic, socioeconomic, and access-related factors that may underlie the association with administrative nationality, before any culturally framed interpretation is adopted; and (4) a structured programme-level analysis of the temporal pattern using screening-process, capacity, and time-to-diagnosis data that were not available for the present study. Routine monitoring of stage at presentation as a performance indicator, with feedback to primary care and screening programme management, would support such work.

## Figures and Tables

**Table 1:** Characteristics of patients with breast cancer in the Aktobe region, stratified by stage grouping, 2015–2025.

Characteristic	Overall (*n* = 2232) ^1^	Stage I–IIa (*n* = 1323) ^1^	Stage IIb–IV (*n* = 909) ^1^	*p*-Value ^2^
**Age at diagnosis, years**	57.0 (12.5)	57.1 (11.7)	56.8 (13.6)	0.7
**Age group, years**				0.002
<40	203 (9.1%)	100 (7.6%)	103 (11.3%)	
40–49	454 (20.3%)	266 (20.1%)	188 (20.7%)	
50–59	574 (25.7%)	357 (27.0%)	217 (23.9%)	
60–69	671 (30.1%)	420 (31.7%)	251 (27.6%)	
≥70	330 (14.8%)	180 (13.6%)	150 (16.5%)	
**Residence**				0.073
Urban	1481 (66.4%)	898 (67.9%)	583 (64.1%)	
Rural	751 (33.6%)	425 (32.1%)	326 (35.9%)	
**Sex**				>0.9
Female	2213 (99.1%)	1312 (99.2%)	901 (99.1%)	
Male	19 (0.9%)	11 (0.8%)	8 (0.9%)	
**Administrative nationality**				0.005
Kazakh	1504 (67.4%)	927 (70.1%)	577 (63.5%)	
Russian	368 (16.5%)	198 (15.0%)	170 (18.7%)	
Other	360 (16.1%)	198 (15.0%)	162 (17.8%)	
**Employment/social status**				0.055
Employed	1005 (45.0%)	612 (46.3%)	393 (43.2%)	
Retired/with a disability	933 (41.8%)	555 (42.0%)	378 (41.6%)	
Unemployed	294 (13.2%)	156 (11.8%)	138 (15.2%)	
**Diagnostic pathway**				<0.001
Clinical examination room detection	124 (5.6%)	83 (6.3%)	41 (4.5%)	
Organised screening	363 (16.3%)	254 (19.2%)	109 (12.0%)	
Patient-initiated/self-referral	1034 (46.3%)	596 (45.0%)	438 (48.2%)	
Other/unspecified	711 (31.9%)	390 (29.5%)	321 (35.3%)	
**Diagnosis period**				<0.001
2015–2017 (pre-expansion)	466 (20.9%)	277 (20.9%)	189 (20.8%)	
2018–2019 (post-expansion, pre-pandemic)	446 (20.0%)	319 (24.1%)	127 (14.0%)	
2020–2022 (pandemic period)	590 (26.4%)	294 (22.2%)	296 (32.6%)	
2023–2025 (post-pandemic period)	730 (32.7%)	433 (32.7%)	297 (32.7%)	

^1^ Mean (SD); n (%); ^2^ Welch’s two-sample *t*-test; Pearson’s chi-squared test. Bold text indicates variable headings. Missing data were 0% for all variables.

## Data Availability

The data analysed in this study are derived from routinely collected administrative and clinical registry records of the Aktobe Regional Oncology Centre. Data are not publicly available due to patient confidentiality provisions; access requests may be directed to the corresponding author subject to institutional data governance approval. The analysis code used to generate all results, tables, and figures is available from the corresponding author on request.

## Supplementary Materials

The following supporting information can be downloaded at https://www.mdpi.com/article/10.3390/medicina62081431/s1, Table S1. Phase-based temporal model. Table S2. Stage distribution by year. Table S3. Global residence-by-year interaction assessed using the likelihood-ratio test. Table S4. Linear residence-by-year interaction estimate. Table S5. Adjusted probabilities by year and residence. Table S6. Model discrimination and calibration statistics. Table S7. Calibration by decile. Table S8. Multinomial stage model: Stage IIb, Stage III, and Stage IV versus Stages I–IIa. Table S9. Sensitivity analysis restricted to female patients. Table S10. Sensitivity analysis using categorical age. Table S11. Sensitivity analysis: outcome restricted to Stage I versus Stages IIa–IV. Figure S1. Stage distribution by year of diagnosis, Aktobe region, 2015–2025. Figure S2. Calibration plot based on deciles of predicted probability, Model B. Figure S3. Receiver operating characteristic curve, Model B.
