# Supplementary material for: Determinants of Early-Stage Breast Cancer Presentation in Western Kazakhstan: A Population-Based Analysis of Urban–Rural Disparities and Diagnostic Pathways (2015–2025)
Source: Medicina (Kaunas). 2026 Jul 23;62(8):1431. doi: 10.3390/medicina62081431 (PMC13515319; doi:10.3390/medicina62081431)
Supplement: Supplementary file 1 [file medicina-62-01431-s001.zip › medicina-4392870-supplementary.pdf]

medicina

Supplementary Materials

Determinants of Early-Stage Breast Cancer Presentation in Western Kazakhstan: A Population-Based Analysis of Urban–Rural Disparities and Diagnostic Pathways (2015–2025)

Dinara Zholmukhamedova, Maiya Taushanova *, Dariusz Walkowiak, Lyudmila Yermukhanova, Laura Danyarova *, Indira Karibayeva, Aizat Aimakhanova, Aizat Seidakhmetova, Anara Tulyayeva

* Correspondence: dr.taushanova@zkmu.kz; la.danyaarova@gmail.com

. Abbreviations: aOR = adjusted odds ratio; RRR = relative risk ratio; CI = confidence interval; AUC = area under the receiver operating characteristic curve; GVIF = generalised variance inflation factor.

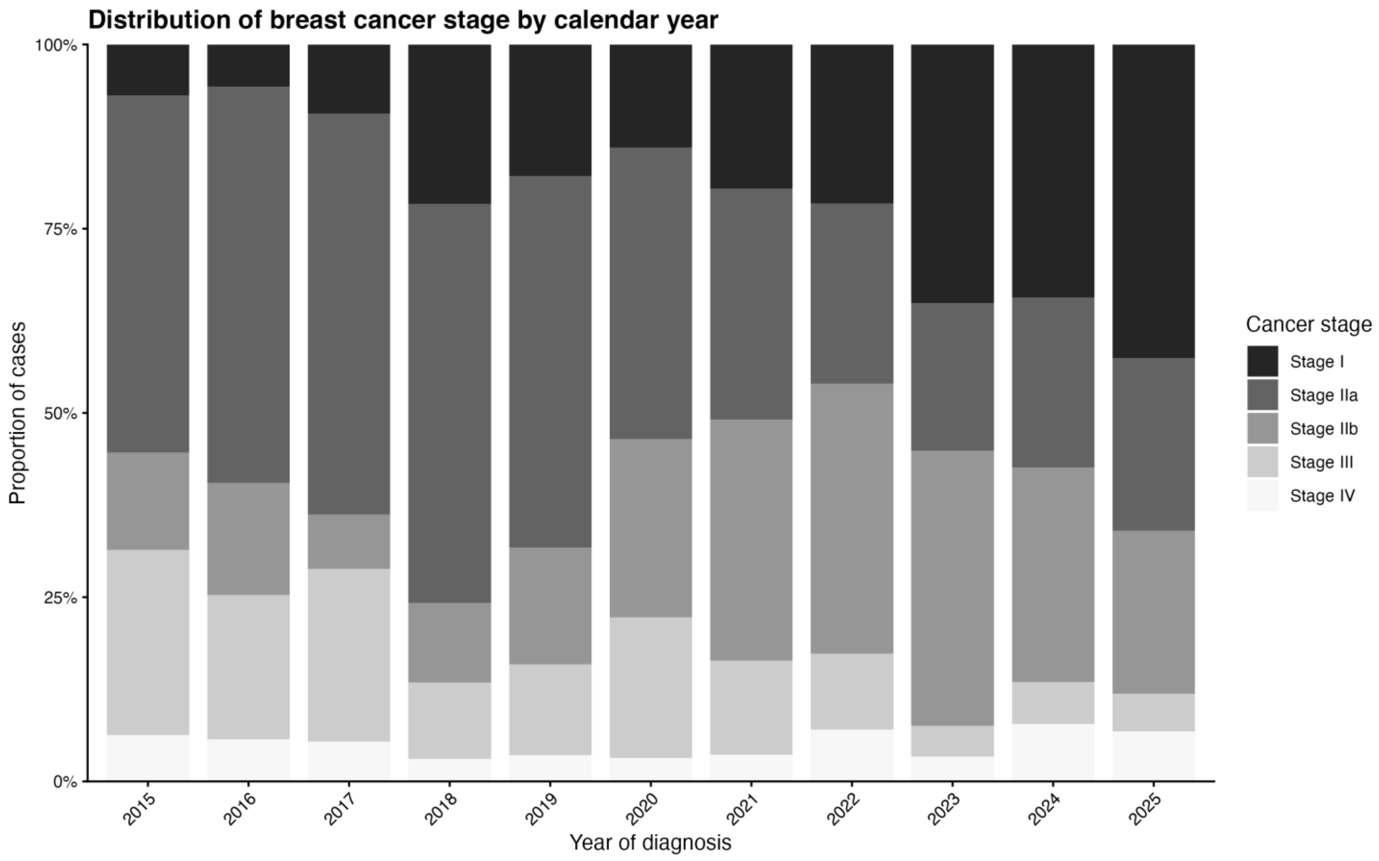

Figure S1. Stage distribution by year of diagnosis, Aktobe region, 2015–2025.

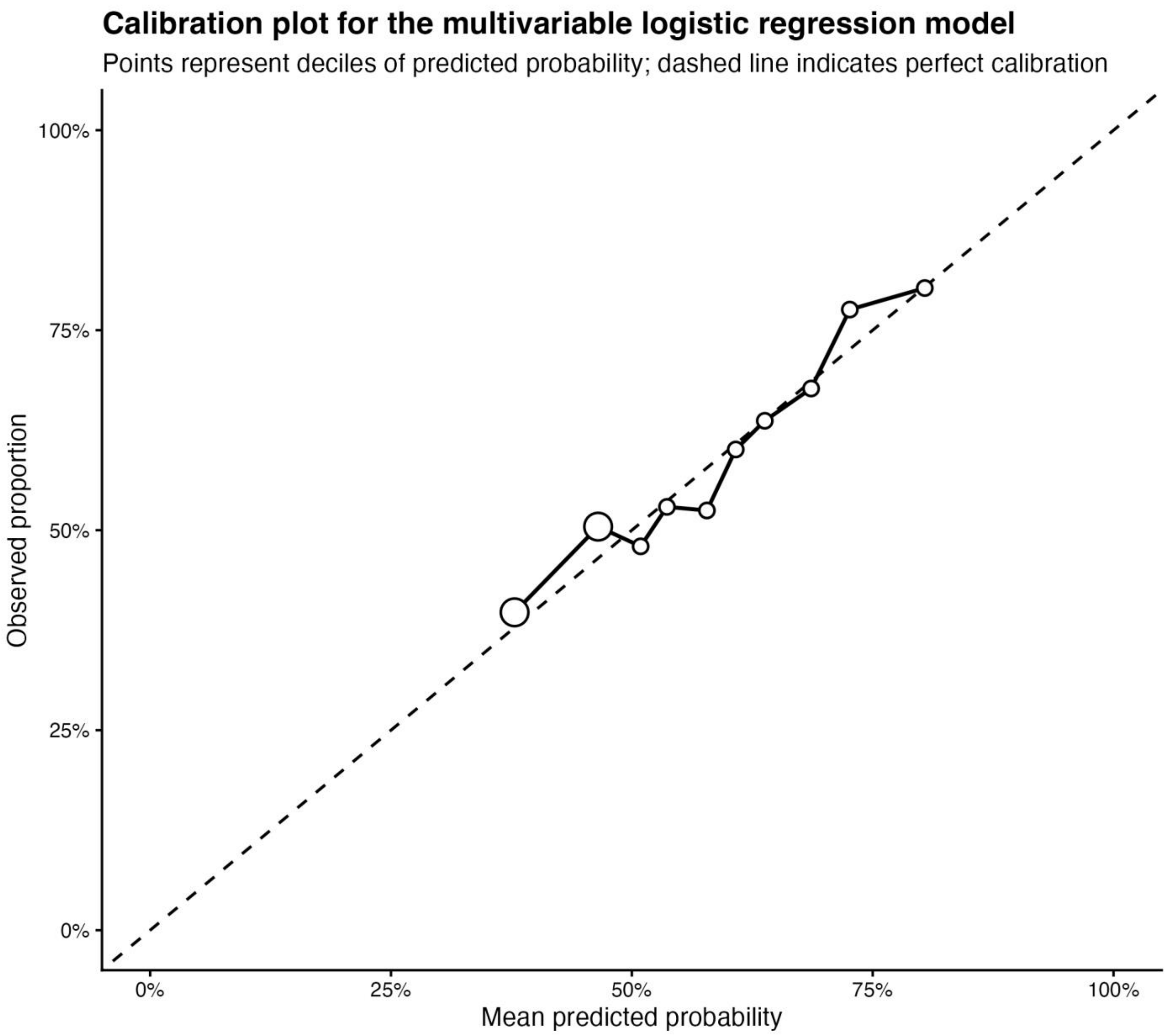

Figure S2. Calibration plot based on deciles of predicted probability, Model B. Points represent deciles of predicted probability; the diagonal line indicates perfect calibration. Hosmer–Lemeshow $\chi^2 = 8.38$, df = 8, p = 0.397.

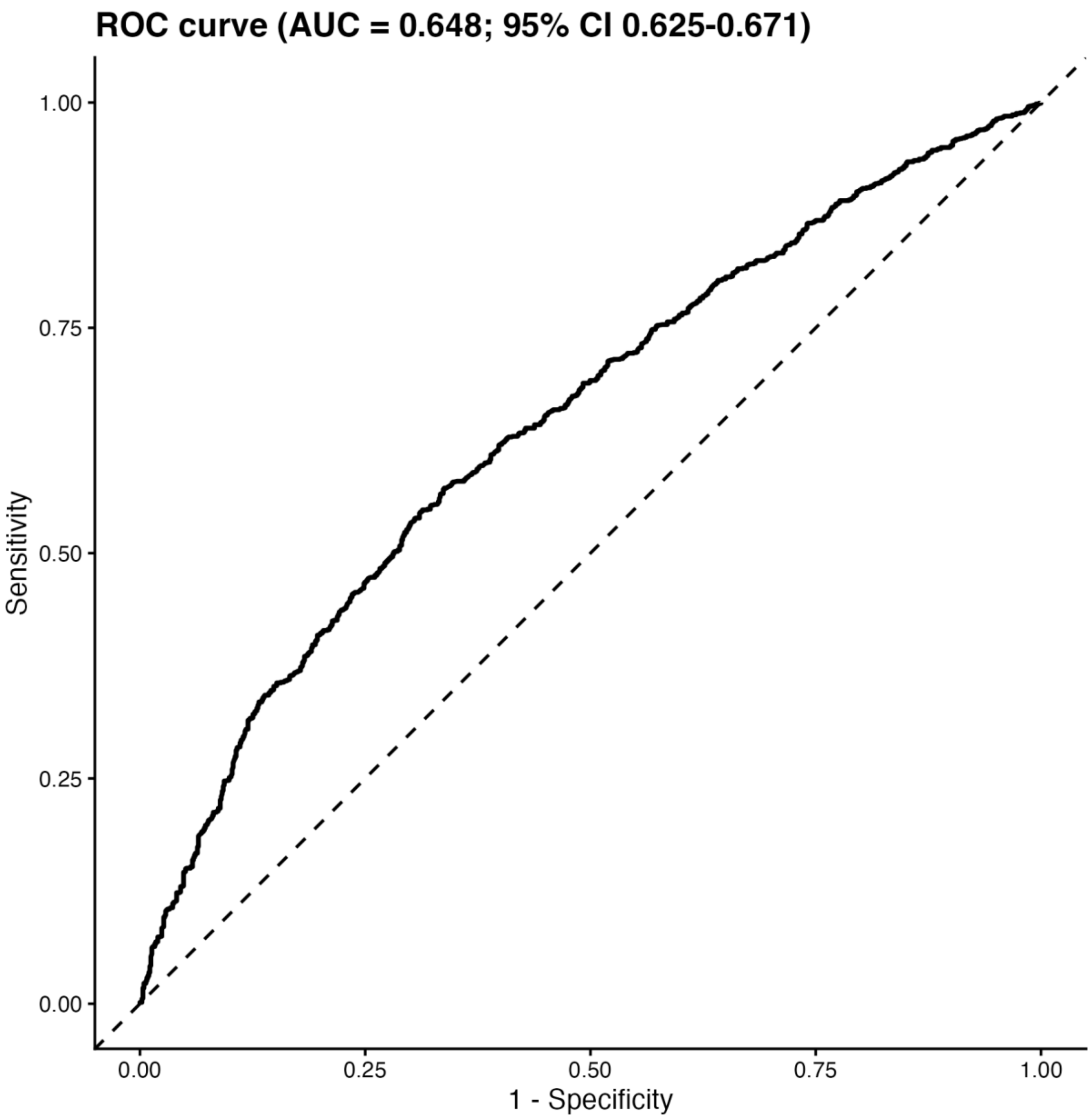

Figure S3. Receiver operating characteristic curve, Model B. AUC = 0.648 (95% CI 0.625–0.671); Brier score = 0.226. The model is presented as a population-level association model and is not suitable for individual-level prediction.

Table S1. Phase-based temporal model: adjusted odds of Stage I–IIa presentation by diagnosis period (reference: 2015–2017).

Diagnosis period	aOR (95% CI)	p-value
Diagnosis period: 2018-2019: post-expansion/pre-pandemic	1.62 (1.22, 2.15)	<0.001
Diagnosis period: 2020-2022: pandemic period	0.54 (0.41, 0.69)	<0.001
Diagnosis period: 2023-2025: post-pandemic period	0.62 (0.46, 0.84)	0.002

Adjusted as in Model B. Reference category: 2015–2017 (pre-expansion period).

Table S2. Stage distribution by year of diagnosis (percentage of cases within each year).

Year of diagnosis	Stage I (%)	Stage IIa (%)	Stage IIb (%)	Stage III (%)	Stage IV (%)
2015	6.9	48.4	13.2	25.2	6.3
2016	5.7	53.8	15.2	19.6	5.7
2017	9.4	54.4	7.4	23.5	5.4

Year of diagnosis	Stage I (%)	Stage IIa (%)	Stage IIb (%)	Stage III (%)	Stage IV (%)
2018	21.6	54.1	10.8	10.3	3.1
2019	17.9	50.4	15.9	12.3	3.6
2020	14	39.5	24.2	19.1	3.2
2021	19.5	31.4	32.7	12.7	3.6
2022	21.6	24.4	36.6	10.3	7
2023	35.1	20	37.4	4.2	3.4
2024	34.3	23	29.1	5.7	7.8
2025	42.6	23.4	22.1	5.1	6.8

Table S3. Global test of effect modification by residence: likelihood-ratio test of the residence-by-calendar-year (categorical) interaction.

Model comparison	Likelihood-ratio χ^2	Degrees of freedom	p-value
Global residence-by-categorical-year interaction	17.52	10	0.064

Table S4. Secondary, compact model: residence-by-calendar-year (linear) interaction.

Interpretation of the interaction term	Interaction OR (95% CI)	p-value
Multiplicative change in the rural-versus-urban odds ratio per calendar year	1.04 (0.98, 1.10)	0.206

The interaction OR expresses the multiplicative change per calendar year in the rural-versus-urban odds ratio for Stage I–IIa presentation.

Table S5. Adjusted annual probability of Stage I–IIa presentation by residence (Model B).

Year of diagnosis	Residence	Adjusted probability of Stage I–IIa, % (95% CI)
2015	Urban	61.9 (51.8–71.0)
2016	Urban	67.4 (58.0–75.7)
2017	Urban	75.0 (65.3–82.7)
2018	Urban	87.2 (80.7–91.8)
2019	Urban	79.7 (72.8–85.1)
2020	Urban	62.7 (52.9–71.5)
2021	Urban	55.9 (47.7–63.8)
2022	Urban	40.5 (32.7–48.7)
2023	Urban	47.0 (39.0–55.2)
2024	Urban	49.5 (41.2–58.0)
2025	Urban	59.3 (50.2–67.8)
2015	Rural	61.9 (49.3–73.1)
2016	Rural	63.9 (50.4–75.5)
2017	Rural	65.7 (53.1–76.5)
2018	Rural	71.3 (60.3–80.3)
2019	Rural	59.5 (48.6–69.4)
2020	Rural	61.5 (46.9–74.3)
2021	Rural	45.6 (33.9–57.8)
2022	Rural	40.3 (28.4–53.6)
2023	Rural	41.1 (30.6–52.6)
2024	Rural	58.2 (45.4–70.0)
2025	Rural	50.1 (38.5–61.7)

Table S6. Discrimination and calibration statistics for Model B.

Performance metric	Value
AUC	0.648
AUC 95% CI lower	0.625

Performance metric	Value
AUC 95% CI upper	0.671
Brier score	0.226
Hosmer-Lemeshow chi-square	8.384
Hosmer-Lemeshow degrees of freedom	8
Hosmer-Lemeshow p-value	0.397

Table S7. Calibration of Model B by decile of predicted probability.

Decile of predicted probability	Patients (n)	Mean predicted probability (%)	Observed proportion of Stage I–IIa (%)
1	224	37.8	39.7
2	224	46.5	50.4
3	223	50.9	48
4	223	53.6	52.9
5	223	57.8	52.5
6	223	60.8	60.1
7	223	63.8	63.7
8	223	68.6	67.7
9	223	72.6	77.6
10	223	80.4	80.3

Table S8. Multinomial logistic regression of stage at diagnosis (reference outcome: Stage I–IIa).

Outcome category (vs. Stage I–IIa)	Characteristic	Adjusted RRR (95% CI)	p-value
Stage IIb	Age at diagnosis (per year)	0.99 (0.98, 1.00)	0.221
Stage IIb	Rural residence (vs urban)	1.23 (0.98, 1.54)	0.073
Stage IIb	Male sex (vs female)	1.19 (0.36, 3.98)	0.779
Stage IIb	Russian nationality (vs Kazakh)	1.41 (1.05, 1.90)	0.022
Stage IIb	Other nationality (vs Kazakh)	1.30 (0.97, 1.74)	0.079
Stage IIb	Retired/with disability (vs employed)	1.08 (0.77, 1.49)	0.661
Stage IIb	Unemployed (vs employed)	1.36 (0.97, 1.92)	0.074
Stage IIb	Organised screening (vs clinical examination room)	0.81 (0.48, 1.37)	0.433
Stage IIb	Patient-initiated/self-referral (vs clinical examination room)	1.38 (0.83, 2.29)	0.222
Stage IIb	Other/unspecified pathway (vs clinical examination room)	1.40 (0.86, 2.30)	0.178
Stage III	Age at diagnosis (per year)	1.00 (0.99, 1.02)	0.617
Stage III	Rural residence (vs urban)	1.74 (1.32, 2.30)	<0.001
Stage III	Male sex (vs female)	1.27 (0.38, 4.21)	0.695
Stage III	Russian nationality (vs Kazakh)	1.49 (1.03, 2.15)	0.032
Stage III	Other nationality (vs Kazakh)	1.32 (0.91, 1.92)	0.143
Stage III	Retired/with disability (vs employed)	1.05 (0.68, 1.62)	0.816
Stage III	Unemployed (vs employed)	1.40 (0.93, 2.11)	0.107
Stage III	Organised screening (vs clinical examination room)	0.79 (0.28, 2.21)	0.648
Stage III	Patient-initiated/self-referral (vs clinical examination room)	4.58 (1.90, 11.04)	<0.001

Outcome category (vs. Stage I–IIa)	Characteristic	Adjusted RRR (95% CI)	p-value
Stage III	Other/unspecified pathway (vs clinical examination room)	2.16 (0.88, 5.30)	0.091
Stage IV	Age at diagnosis (per year)	1.00 (0.98, 1.02)	0.903
Stage IV	Rural residence (vs urban)	1.08 (0.70, 1.67)	0.738
Stage IV	Male sex (vs female)	0.00 (0.00, 0.00)	<0.001
Stage IV	Russian nationality (vs Kazakh)	2.00 (1.21, 3.30)	0.007
Stage IV	Other nationality (vs Kazakh)	1.56 (0.91, 2.69)	0.105
Stage IV	Retired/with disability (vs employed)	1.12 (0.59, 2.13)	0.735
Stage IV	Unemployed (vs employed)	2.22 (1.25, 3.93)	0.006
Stage IV	Organised screening (vs clinical examination room)	0.06 (0.01, 0.30)	<0.001
Stage IV	Patient-initiated/self-referral (vs clinical examination room)	3.95 (1.49, 10.47)	0.006
Stage IV	Other/unspecified pathway (vs clinical examination room)	0.86 (0.35, 2.12)	0.746

Adjusted for age, sex, residence, administrative nationality, employment/social status, diagnostic pathway, and calendar year (categorical). Year-specific coefficients are omitted for readability. The Stage IV estimate for male sex is unstable owing to complete separation (no male Stage IV cases) and should not be interpreted.

Table S9. Sensitivity analysis: analytic sample restricted to female patients (n = 2,213).

Characteristic	aOR (95% CI)	p-value
Age at diagnosis (per year)	1.00 (0.99, 1.01)	0.503
Rural residence (vs urban)	0.74 (0.61, 0.89)	0.002
Russian nationality (vs Kazakh)	0.67 (0.52, 0.85)	0.001
Other nationality (vs Kazakh)	0.74 (0.58, 0.95)	0.017
Retired/with disability (vs employed)	0.91 (0.69, 1.21)	0.523
Unemployed (vs employed)	0.68 (0.52, 0.90)	0.007
Organised screening (vs clinical examination room)	1.47 (0.92, 2.36)	0.108
Patient-initiated/self-referral (vs clinical examination room)	0.44 (0.28, 0.69)	<0.001
Other/unspecified pathway (vs clinical examination room)	0.71 (0.46, 1.09)	0.120

Adjusted as in Model B. Year-specific coefficients are omitted for readability.

Table S10. Sensitivity analysis: age modelled as a categorical variable.

Characteristic	aOR (95% CI)	p-value
Age group (40-49)	1.29 (0.91, 1.83)	0.148
Age group (50-59)	1.44 (1.03, 2.02)	0.035
Age group (60-69)	1.66 (1.06, 2.61)	0.026
Age group (70+)	1.38 (0.84, 2.28)	0.208
Rural residence (vs urban)	0.74 (0.61, 0.89)	0.001
Male sex (vs female)	0.95 (0.37, 2.44)	0.914
Russian nationality (vs Kazakh)	0.67 (0.53, 0.86)	0.001
Other nationality (vs Kazakh)	0.75 (0.59, 0.96)	0.021
Retired/with disability (vs employed)	0.86 (0.60, 1.22)	0.388
Unemployed (vs employed)	0.70 (0.53, 0.93)	0.014
Organised screening (vs clinical examination room)	1.40 (0.87, 2.25)	0.167
Patient-initiated/self-referral (vs clinical examination room)	0.47 (0.30, 0.73)	<0.001
Other/unspecified pathway (vs clinical examination room)	0.72 (0.47, 1.10)	0.129

Adjusted as in Model B, with continuous age replaced by age groups (reference: <40 years). Year-specific coefficients are omitted for readability.

Table S11. Sensitivity analysis: outcome restricted to Stage I versus Stage IIa–IV.

Characteristic	aOR (95% CI)	p-value
Age at diagnosis (per year)	1.00 (0.99, 1.01)	0.808
Rural residence (vs urban)	0.98 (0.79, 1.23)	0.883
Male sex (vs female)	1.87 (0.64, 5.46)	0.252
Russian nationality (vs Kazakh)	0.74 (0.55, 1.01)	0.059
Other nationality (vs Kazakh)	0.66 (0.48, 0.90)	0.009
Retired/with disability (vs employed)	1.15 (0.83, 1.60)	0.386
Unemployed (vs employed)	0.79 (0.54, 1.16)	0.233
Organised screening (vs clinical examination room)	1.44 (0.89, 2.31)	0.136
Patient-initiated/self-referral (vs clinical examination room)	0.55 (0.34, 0.89)	0.015
Other/unspecified pathway (vs clinical examination room)	0.71 (0.45, 1.13)	0.149

Adjusted as in Model B. Year-specific coefficients are omitted for readability.
